# WITHIN- AND BETWEEN-PERSON DIFFERENCES IN ACTIVITY FACTOR STRUCTURE: RESULTS FROM AN EMA STUDY

**DOI:** 10.1093/geroni/igad104.2215

**Published:** 2023-12-21

**Authors:** Allison Bielak, Jacqueline Mogle, Martin Sliwinski

**Affiliations:** Colorado State University, Fort Collins, Colorado, United States; Clemson University, Clemson, South Carolina, United States; Pennsylvania State University, State College, Pennsylvania, United States

## Abstract

Analyses of activity participation scales are typically based on single time points and resulting factors focus on differences between participants. However, as real-world activity engagement varies at more micro-timescales, these analyses provide little insight into how activities cluster together within a person across moments or days. Ecological momentary assessment (EMA) studies capture within-person fluctuations in activity engagement and allow us to compare within-person variability to that observed across persons. Using multilevel factor analysis we examined the factor structure of activity participation within-persons (i.e., across days) and between participants. Using tablet-based assessments, 81 adults aged 41 to 94 years (M=61.26, SD=12.12) reported the activities they completed in the past 3-4 hours 5 times per day (4 at semi-randomly scheduled intervals and 1 at bedtime) for 14 days. Multilevel factor analysis simultaneously computed both intra-individual factors (within-person structure of activity in daily life) and inter-individual factors (between-person structure of activity engagement). A solution of 4 within-person and 4 between-person factors provided the best model fit, with three common factors across levels: 1) cognitive (e.g., read, write, computer tasks); 2) social (e.g., events, mentoring, providing care); and 3) passive (e.g., TV, games) factors. There were notable differences in the fourth factor however. Although there are similarities, the factor structure of activity participation between individuals is different than factors describing activity participation within persons from day to day. Researchers should be aware that common between-person activity factors will not unilaterally fit EMA within-person data and should conduct additional preparatory factor analyses.

